# Variations in DEPDC5 gene and its association with chronic hepatitis C virus infection in Saudi Arabia

**DOI:** 10.1186/s12879-014-0632-y

**Published:** 2014-12-31

**Authors:** Mashael R Al-Anazi, Sabine Matou-Nasri, Ayman A Abdo, Faisal M Sanai, Mohammed Q Khan, Ali Albenmousa, Hamad I Al-Ashgar, Nisreen Z Khalaf, Mohammed N Al-Ahdal, Ahmed A Al-Qahtani

**Affiliations:** Department of Infection and Immunity, Research Center, King Faisal Specialist Hospital & Research Center, Riyadh, Saudi Arabia; King Abdullah International Medical Research Center, National Guard Health Affairs, Riyadh, Saudi Arabia; Department of Medicine, College of Medicine, King Saud University, Riyadh, Saudi Arabia; Gastroenterology Section, Department of Medicine, King Abdulaziz Medical City, Jeddah, Saudi Arabia; Department of Medicine, King Faisal Specialist Hospital & Research Center, Riyadh, Saudi Arabia; Department of Gastroenterology & Hepatology, Prince Sultan Military Medical City, Riyadh, Saudi Arabia; Department of Microbiology and Immunology, Alfaisal University School of Medicine, Riyadh, Saudi Arabia; Department of Pathology and Laboratory Medicine, King Faisal Specialist Hospital and Research Center, Riyadh, Saudi Arabia; Liver Disease Research Center, King Saud University, Riyadh, Saudi Arabia

## Abstract

**Background:**

Variations at DEPDC5 gene have been recently reported as genetic markers associated with hepatocellular carcinoma (HCC) progression in chronic HCV-infected patients. This study was conducted to assess the association of DEPDC5 variants with advanced liver cirrhosis and HCC development among chronic HCV-infected patients in Saudi Arabian population.

**Methods:**

Six-hundred and one HCV-infected patients were genotyped for DEPDC5 polymorphisms (rs1012068 and rs5998152), in comparison with 592 non-infected healthy control subjects. The allelic frequency and genotype distribution of both DEPDC5 polymorphisms were determined followed by haplotype frequency estimation and multiple logistic regression analysis.

**Results:**

The frequency of the risk alleles of both rs1012068 and rs5998152 was shown to be more in healthy control subjects than in patients (p = 0.0001, OR = 0.704, CI = 0.591-0.839; p = 0.002, OR = 0.761, CI = 0. 0.639-0.907, respectively). Also, our results revealed that GT for SNP rs1012068 (OR =1.715; 95% CI 1.132-2.597; p = 0.0104) and CT for SNP rs5998152 (OR = 1.932; 95% CI 1.276-2.925; p = 0.0017) showed significant association with development of cirrhosis compared with the GG and CC genotypes, respectively. The data also revealed that subjects with the T allele of both SNPs appeared to have a lower susceptibility to HCV-related cirrhosis/HCC than those with the G allele of rs1012068 (p = 0.038, OR = 1.353, 95 % CI 1.017-1.800) and C allele of rs5998152 (p = 0.043, OR = 1.342, 95 % CI 1.010-1.784). Haplotype analysis showed that a combination of T-T alleles of rs1012068 and rs5998152 was significantly associated with liver cirrhosis (frequency = 71.3% and p = 0.027) and with cirrhosis/HCC (frequency = 71.4% and P = 0.045). Also, multiple logistic regression analysis showed that rs5998152 (OR = 2.844, 95% CI 1.333-6.069 and p = 0.007), rs1012068 (OR = 2.793, 95% CI 1.316-5.928 and p = 0.010), age (OR = 1.029, 95% CI 1.001-1.057 and p = 0.041) and HCV genotypes (OR = 0.247, 95% CI 0.097-0.630 and p = 0.003) were independently associated with chronicity of HCV infection.

**Conclusion:**

Genetic variations in DEPDC5 gene region may influence HCV-associated liver cirrhosis and/or HCC development.

## Background

Hepatitis C virus (HCV) is a single-stranded RNA virus that belongs to the Flaviviridae family, genus *Hepacivirus*. It is an enveloped and a non-cytopathic virus. Over 180 million people are infected with HCV worldwide including 94.6 million people in the Western Pacific and Southeast Asia regions, with an estimated infection rate of more than 200 million people by the year 2020 [1],[2]. More than 30% of acute infections are asymptomatic with the possibility of clearance by the natural immune defenses while 70-85% of patients will develop chronic HCV infection [3],[4]. About 15% of chronically infected patients develop cirrhosis and, eventually, 5% of these cirrhotic patients may go on to develop hepatocellular carcinoma (HCC) within 5 years [5],[6] .

HCV proteins, particularly Core and NS5A, induce endoplasmic reticulum stress causing liver injury and inflammation. This is followed by the over-production and accumulation of extracellular matrix proteins induced by transforming growth factor (TGF)- β, which leads to the formation of fibrosis and distorts the hepatic architecture [7],[8]. The molecular pathogenesis of HCV-associated HCC development has been reported to be mainly caused by HCV core-activated TGF- β that acts as a tumor promoting agent through its ability to bind SMAD3, the major transcription factor of TGF-β signaling [9],[10].

Recent studies have identified several risk factors for the severity of the outcome of HCV infection including older age, male gender, viral genotype, the virus mutation rate, co-infection with other viruses (such as HIV and HBV), metabolic factors (such as obesity, insulin resistance, and steatosis), and high alcohol and tobacco consumptions [11]. In addition, host genetic polymorphisms in several genes (such as HLA-DRB1, TNF-α, IL-10, TGF-β1 and p53) have been directly linked to increased severity of HCV-associated diseases [12]-[14].

Recently, there is a growing interest in the identification of genetic markers influencing the risk of developing HCV-associated cirrhosis and HCC [15]. Using high-throughput genomic technology, a genome-wide association study (GWAS) has revealed one intronic single nucleotide polymorphism (SNP) from the DEPDC5 (Dishevelled, Egl-10 and Pleckstrin domain-containing 5) region that was strongly associated with the risk of HCC progression in Japanese patients chronically infected with HCV [16]. DEPDC5 gene has been localized to the long arm of chromosome 22, 22q12.3 and it encodes a cytoplasmic protein which has been recently shown to have a central role in focal epilepsy, a neurological disorder [17]. The function of the DEPDC5 has not been defined yet; however, DEPDC5 protein has been demonstrated to block the effect of mammalian target of rapamycin (mTOR), a multi-functional protein involved in many cellular systems including inflammation, cell growth and tumorigenesis including hepatocarcinogenesis [18]-[20].

In this study we aimed to determine the link between DEPDC5 variations, namely SNPs rs1012068 and rs5998152 (located 2.7 kb upstream of rs1012068), and the risk of developing HCC in chronic HCV-infected patients. The association between liver cirrhosis and these SNPs was also examined as HCC almost exclusively occurs in the context of cirrhosis in HCV patients and it is considered as a pre-neoplastic condition for the development of HCC [21].

## Methods

### Patients

From August 2007 to August 2010, 601 Saudi patients infected with HCV (anti-HCV serology positive with a detectable HCV RNA) were recruited from three major hospitals in Riyadh city, including King Faisal Specialist Hospital & Research Center, Riyadh Military Hospital, and King Khalid University Hospital. The patients were grouped into three categories based on disease severity as follows: **Group I**: chronic HCV patients who were diagnosed on the basis of the presence of antibodies against HCV (anti HCV) and serum HCV RNA for more than six months. This group includes patients with normal, elevated and fluctuating levels of alanine aminotransferase (ALT). However, none of these patients showed any biochemical, clinical, radiological or histological evidence of cirrhosis (n = 450), **Group II:** patients having cirrhosis (n = 124) and **Group III:** cirrhotic patients with HCC (n = 27). Blood samples were also collected from 592 normal healthy subjects who volunteered to participate in this study. Control subjects were characterized by the absence of any known serological marker for HCV, HCV RNA or any evidence of liver disease. For non-histological diagnosis of cirrhosis, a fibroscan consistent with cirrhosis [22] or a combination of any four of the following criteria was required: platelet count <100× 10^9^/L, presence of esophageal varices, imaging features consistent with cirrhosis, albumin level less than 30 g/L, INR more than 1.4 and bilirubin >30 μmol/L [23],[24]. The diagnosis of HCC was based upon established guidelines criteria [25]. Briefly, enhancement of a liver lesion during the arterial phase and contrast washout during the portal phase, in patients with background cirrhosis was considered diagnostic of HCC. Trucut biopsy was performed only where considerable doubt existed after imaging studies. To avoid many confounding factors that could cause liver abnormalities, patients with the following clinical conditions were excluded from the study: significant alcohol ingestion, co-infection with HBV and/or HIV and other causes of liver disease such as Wilson’s disease, hemochromatosis and autoimmune hepatitis.

### Ethics statement

The study was approved by the institutional review boards of all participating hospitals, and conducted in accordance with the Helsinki Declaration of 1975. Informed consents were obtained from all patients and healthy control subject.

### Genotyping of HCV

Nucleic acids were extracted from sera using the QIAmp MinElute Virus Spin Kit (QIAGEN, Santa Clarita, California, USA) following the manufacturer’s instructions. Genotyping was carried out using INNO-LiPA HCV II (Innogenetics NV, Ghent, Belgium) according to the manufacturer’s instructions.

### Genotyping of DEPDC5 SNPs

Genomic DNA was isolated from buffy coat of the HCV patients and control subjects using Gentra Pure Blood kit (Qiagen, Hilden, Germany) according to manufacturer’s recommendations. Real-time PCR TaqMan allelic discrimination assays were purchased from Applied Biosystems (Applied Biosystems, Foster City, CA). They were used to perform genotyping of SNPs rs1012068 (assay ID: C_27100398_10) and rs5998152 (assay ID: C_2456027_10). These assays were designed to utilize SNP specific primers and two allele-specific TaqMan® minor groove binder (MGB) probes. For SNP rs1012068, one of the probes was specific for G allele and was labeled with the reporter dye VIC and the other was specific for T allele and was labeled with the reporter dye FAM. For SNP rs5998152, one of the probes was specific for C allele and labeled with the reporter dye VIC and the other was specific for T allele and was labeled with the reporter dye FAM.

The reactions were performed in 96-well plates in a total reaction volume of 25 μl using 20 ng of genomic DNA. The mixture contained 900 nM of each primer and 200 nM of each labeled probe and TaqMan universal PCR master mix. The plates were subjected to the following cycling parameters: 95°C for 10 minutes, followed by 40 cycles of 92°C for 15 seconds for denaturation and 60°C for 1.5 minutes for annealing and extension. Genotypes were assessed by the TaqMan allele-specific assay method using the ABI Prism® 7500 Sequence Detection System software, according to the manufacturer’s protocols (Applied Biosystems, Foster City, CA).

### Statistical analysis

Statistical analysis was performed using SPSS version 20.0 (SPSS Inc., Chicago, IL, USA) and HaploView version 4.2. The association between the DEPDC5 SNPs and the disease status were expressed in odds ratio (OR) and their 95% confidence intervals (CI). A p <0.05 was considered to be statistically significant. The SNPs were tested for Hardy–Weinberg equilibrium (HWE) using the program (http://ihg.gsf.de/cgi-bin/hw/hwa1.pl). A cut-off p-value of 0.05 was set for HWE and SNPs were excluded from the study only if minor allele frequency (MAF) of 1% was found.

## Results

The demographic and clinical characteristics of the study subjects, HCV-infected patients (601) and control subjects (592), are presented in Table 1. Logistic regression analysis confirmed that older age and male gender were significantly associated with high risk of HCV chronic infection. Among the host factors known as predictive indicators of the progression of end-stage liver disease, body mass index (BMI), platelet count, metabolic activities (i.e. ALT, ASP and ALP) were assessed in HCV-infected patients. End-stage liver disease was found to be significantly influenced by the drop of platelet count and elevation of AST level, while, BMI, ALT and ALP levels were not significantly different among the groups.

**Table 1:** **Demographic and clinical characteristics of HCV-infected patients and healthy control subjects**

Variable	Chronic HCV	Liver cirrhosis	HCC	Healthy control	pvalue ^a^
Age (yrs)	50.9 ± 14.98	58.30 ± 11.86	63.23 ± 10.70	30.79 ± 8.90	<0.0001
Male gender (%)	233 (51.4%)	61 (50%)	12 (46.2%)	550 (96.7%)	<0.0001
BMI (Kg/m^2^)*	29.2 (25.67-33.39)	30.06 (26.22-33.06)	25.99 (21.89-31.39)		0.804
Platelets (10^9^/L)*	230 (178.75-282.25)	163.5 (108.50-205.50)	104.00 (51.50-139.00)		<0.0001
ALT (U/L)**	68.973 ± 57.99	78.66 ± 50.76	114.33 ± 31.78		0.738
AST (U/L)**	43.54 ± 33.30	72.67 ± 48.81	91.33 ± 24.58		<0.0001
ALP (U/L)**	113.37 ± 78.91	133.66 ± 90.04	147.00 ± 96.70		0.072
DM2					
Overall (30.8%)	134 (72.4%)	44 (23.8%)	7 (3.8%)		0.693
HCV genotypes					
4 (357)	249 (70%)	86 (24%)	22 (6%)		<0.0001
1 (148)	115(77.7%)	30 (20.3%)	3 (2%)		
Others¶¶ (96)	89 (92.7%)	6 (6.3%)	1 (1%)		
HCV RNA (Log10)*	5.85 (4.81-6.54)	5.63 (4.86-6.32)	5.98 (2.79-7.24)		0.552

*Values are expressed as median interquartile range (25th - 75th), **Values are expressed as mean ± SD.

p^a^: nonparametric test and one way Anova for continuous data and Chi square test for categorical data.

¶¶ includes genotypes 2, 3 and untypable genotypes. *Abbreviations*: *HCV* hepatitis C virus, *HCC* hepatocellular carcinoma, *ALT* alanine aminotransferase, *AST* aspartate aminotransferase, *ALP* Alkaline Phosphatase, *BMI* body mass index; DM2: diabetes mellitus.

The distribution of HCV genotypes was determined in these patients. Our results revealed that the majority of patients were infected with genotype 4 (357/601, 59.4%), followed by genotype 1 (148/601, 24.63%). Other samples belonged to genotypes 2 and 3 in addition to few samples that could not be typed by the method used (96/601, 15.97%) (Table 1).

Additionally, the prevalence of diabetes mellitus type 2 (DM2) was determined in the patients included in this study. Our results showed that the overall incidence of DM2 was 30.8% (185/601). One-hundred and thirty four (72.4%) chronic HCV patients were found to have DM2, while, DM2 incidence was 23.8% (44/185) and 3.8% (7/185) among patients with cirrhosis and HCC, respectively (Table 1).

The distribution of the genotypes and allele frequency of the two SNPs (rs1012068 and rs5998152) were studied in chronic HCV-infected patients in comparison with healthy control subjects. Genotype distribution of each SNP was in accordance with Hardy-Weinberg equilibrium (HWE) in both control and chronic HCV-infected groups (Table 2).

**Table 2:** **Heterozygosity and minor allele frequency of the DEPDC5 between control subjects and chronic HCV-infected patients**

Name	Position	ObsHET	PredHET	HWpval	MAF	Alleles
rs1012068	32265903	0.448	0.424	0.059	0.306	T:G
rs5998152	32263162	0.437	0.421	0.2293	0.301	T:C

First, we checked the allele and genotype frequency of both SNPs in patients clinically proven to carry the virus compared to non-infected healthy control group (Table 3). When compared to the reference TT genotype, both GG (OR = 0.414; 95% CI 0.264-0.651; p = <0.0001) and GT (OR = 0.743; 95% 0.586-0.943; p = 0.014) for SNP rs1012068 showed a significant inverse association with chronic HCV infection as they were distributed more in the control subjects than HCV-infected patients. Likewise, for SNP rs5998152 the TT was selected as reference genotype and compared to the other genotypes. There was a significant difference in distribution for both CC (OR = 0.544; 95% CI 0.352-0.840; p = 0.006) and CT (OR = 0.772; 95% CI 0.608-0.979; p = 0.033). Under the recessive model, both variants were significantly associated with HCV infection; rs1012068 (OR = 2.086; 95% CI 1.348-3.228; p = 0.0008) and rs5998152 (OR = 1.625; 95% CI 1.068-2.474; p = 0.022) (Table 3).

**Table 3:** **DEPDC5 genetic variations between chronic HCV-infected patients and control group**

SNPs	Genotype distribution/allele frequency	Controls (592)	Patients (601)	OR (95% C.I.)	χ ^2^	p value
rs1012068	GG	64 (10.8%)	33 (5.5%)	0.414 (0.264-0.651)	15.200	<0.0001
	GT	278 (47%)	257 (42.8%)	0.743 (0.586-0.943)	6.010	0.014
	TT	250 (42.2%)	311 (51.7%)	Ref		
	GG + GT.VS.TT			0.682 (0.542-0.857)	10.840	<0.001
	TT + GT.VS.GG			2.086 (1.348-3.228)	11.300	0.0008
	Allele					
	G	406 (34.3%)	323 (26.9%)	0.704 (0.591-0.839)	15.470	<0.0001
	T	778 (65.7%)	879 (73.1%)			
rs5998152	CC	60 (10%)	39 (6.5%)	0.544 (0.352-0.840)	7.670	0.006
	CT	271 (46%)	250 (41.6%)	0.772 (0.608-0.979)	4.570	0.033
	TT	261 (44%)	312 (51.9%)	Ref		
	CC + CT.VS.TT			0.730 (0.582-0.917)	7.320	0.007
	TT + CT.VS.CC			1.625 (1.068-2.474)	5.210	0.022
	Allele					
	C	391 (33%)	328 (27.3%)	0.761 (0.639-0.907)	9.320	0.002
	T	793 (67%)	874 (72.7%)			

OR: odds ratio; CI: confidence interval.

In order to assess the influence of DEPDC5 polymorphism on the risk of progression to cirrhosis or HCC, the genotype distribution and allele frequency of both DEPDC5 SNPs were studied between chronic HCV-infected patients and cirrhotic patients (Table 4). Genotype GT for SNP rs1012068 (OR = 1.715; 95% CI 1.132-2.597; p = 0.010) and CT for SNP rs5998152 (OR = 1.932; 95% CI 1.276-2.925; p = 0.002) showed a significant association with development of cirrhosis (Table 4). Furthermore, there was a significant difference in distribution and frequency for the alleles of both SNPs (G *vs* T for rs1012068 with an OR = 1.456; 95% CI 1.074-1.974; p = 0.015) and (C *vs* T for rs5998152 with an OR = 1.397; 95% CI 1.030-1.894; p = 0.031) (Table 4).

**Table 4:** **DEPDC5 genetic variations between chronic HCV-infected patients (Group 1) and cirrhotic HCV-infected patients (Group 2)**

SNPs	Genotype/allele distribution	Chronic (450)	Cirrhosis (124)	OR (95% C.I.)	χ ^2^	p value
rs1012068	GG	24 (5.3%)	9 (7.3%)	1.809 (0.794-4.121)	2.040	0.154
	GT	180 (40%)	64 (51.6%)	1.715 (1.132-2.597)	6.570	0.010
	TT	246 (54.7%)	51 (41.1%)	Ref		
	GG + GT.VS.TT			1.726 (1.154-2.583)	7.140	0.008
	TT + GT.VS.GG			0.720 (0.326-1.591)	0.660	0.415
	Allele					
	G	228 (25.3%)	82 (33%)	1.456 (1.074-1.974)	5.900	0.015
	T	672 (74.7%)	166 (67%)			
rs5998152	CC	30 (6.7%)	7 (5.7%)	1.157 (0.481-2.782)	0.110	0.744
	CT	172 (38.2%)	67 (54%)	1.932 (1.276-2.925)	9.860	0.002
	TT	248 (55.1%)	50 (40.3%)	Ref		
	CC + CT.VS.TT			1.817 (1.213-2.722)	8.520	0.004
	TT + CT.VS.CC			1.194 (0.511-2.787)	0.170	0.682
	C	232 (25.8%)	81(33%)	1.397 (1.030-1.894)	4.650	0.031
	T	668 (74.2%)	167(67%)			

OR: odds ratio; CI: confidence interval.

Likewise, the genotype distribution and allele frequency of both SNPs were analyzed between chronically infected patients and cirrhotic and HCC patients combined (Cirr/HCC). A highly significant association was observed for the heterozygous genotypes for both SNPs as both were more frequent in Cirr/HCC than chronic patients. GT genotype of rs1012068 exhibited an OR = 1.619; 95% 1.105-2.372; p = 0.013, while, CT genotype of rs5998152 showed an OR 1.757; 95% CI 1.197-2.579; p = 0.004 (Table 5). The risk alleles G and C for SNP rs1012068 (OR of 1.353; 95% CI 1.017-1.800, p = 0.038) and rs5998152 (OR = 1.342; 95% CI 1.010-1.784; p = 0.043), respectively, were found to be associated with development of Cirr/HCC (Table 5). The T allele for SNP rs1012068 showed a significant association under the dominant model (OR =1.595; 95% CI 1.100-2.314; p = 0.013). Similarly, the T allele of rs5998152 showed an association under the dominant model (OR = 1.669; 95% CI 1.150-2.422; p = 0.007).

**Table 5:** **DEPDC5 genetic variations among chronic HCV-infected patients (Group 1) and cirrhotic HCV-infected patients diagnosed with HCC (Group 3)**

SNPs	Genotype/allele distribution	Chronic (450)	Cirrhosis + HCC (151)	OR (95% C.I.)	χ ^2^	p value
rs1012068	GG	24 (5.3%)	9 (6%)	1.419 (0.629-3.201)	0.720	0.397
	GT	180 (40%)	77 (51%)	1.619 (1.105-2.372)	6.160	0.013
	TT	246 (54.7%)	65 (43%)	Ref		
	GG + GT.VS.TT			1.595 (1.100-2.314)	6.110	0.013
	TT + GT.VS.GG			0.889 (0.404-1.957)	0.090	0.769
	Allele					
	G	228 (25.3%)	95 (31.5%)	1.353(1.017-1.800)	4.310	0.038
	T	672 (74.7%)	207 (68.5%)			
rs5998152	CC	30 (6.7%)	9 (6%)	1.163 (0.526-2.572)	0.140	0.701
	CT	172 (38.2%)	78 (52%)	1.757 (1.197-2.579)	8.390	0.004
	TT	248 (55.1%)	64 (42%)	Ref		
	CC + CT.VS.TT			1.669 (1.150-2.422)	7.340	0.007
	TT + CT.VS.CC			1.127 (0.522-2.431)	0.090	0.760
	Allele					
	C	232 (25.8%)	96 (32%)	1.342 (1.010-1.784)	4.120	0.043
	T	668 (74.2%)	206 (68%)			

HCC: hepatocellular carcinoma; OR: odds ratio; CI: confidence interval.

Haplotype analysis for the two DEPDC5 SNPs was performed between chronic HCV-infected patients compared to healthy control subjects. Haplotype combinations and their frequency of occurrence are represented in Table 6. Two out of the three blocks were found to be significant, block TT (frequency = 0.684 and p = 0.001) and CG (frequency = 0.293 and p = 0.0004). Additionally, two out of four blocks were found to be significant when chronic patients were compared to patients with cirrhosis. The two blocks are TT (frequency = 0.713 and p = 0.0267) and CG (frequency = 0.261 and p = 0.032) (Table 7). However, in regard to the association haplotype analysis between chronic HCV-infected patients and Cirr/HCC patients, only one haplotype out of four carrying the dominant genotype TT was significant with a frequency reaching 0.714 and p = 0.045 (Table 8).

**Table 6:** **Haplotype frequencies of the DEPDC5 between healthy control and chronic HCV-infected groups**

Block	Freq.	Chronic HCV, healthy control ratio	Chronic HCV, healthy control frequencies	Chi square	p value
TT	0.684	869.6:348.4, 772.8:411.2	0.714, 0.653	10.402	0.0013
CG	0.293	317.3:900.7, 385.8:798.2	0.261, 0.326	12.390	0.0004
TG	0.014	14.6:1203.4, 20.2:1163.8	0.012, 0.017	1.080	0.2987

**Table 7:** **Haplotype frequencies of the DEPDC5 between chronic HCV-infected patients and cirrhotic HCV-infected patients**

Block	Freq.	Liver cirrhosis, HCV patient ratio	Liver cirrhosis, HCV patient frequencies	Chi square	p value
TT	0.713	162.9:85.1, 667.6:248.4	0.657, 0.729	4.913	0.027
CG	0.261	77.9:170.1, 226.2:689.8	0.314, 0.247	4.593	0.032
CT	0.013	3.1:244.9, 11.7:904.3	0.012, 0.013	0.004	0.951
TG	0.013	4.1:243.9, 10.5:905.5	0.016, 0.012	0.370	0.543

**Table 8:** **Haplotype frequencies of the DEPDC5 between chronic HCV-infected patients and cirrhotic HCV-infected patients diagnosed with HCC**

Block	Freq.	Liver cirrhosis + HCC, HCV patients ratio	Liver cirrhosis + HCC, HCV patients frequencies	Chi square	p value
TT	0.714	201.9:100.1, 667.5:248.5	0.669, 0.729	4.017	0.045
CG	0.26	90.9:211.1, 226.1:689.9	0.301, 0.247	3.470	0.063
CT	0.014	5.1:296.9, 11.8:904.2	0.017, 0.013	0.253	0.615
TG	0.012	4.1:297.9, 10.5:905.5	0.013, 0.011	0.074	0.785

To adjust for other risk factors that could contribute to disease progression associated with HCV infection, a multivariate logistic regression analysis was performed, which showed that rs5998152 (OR = 2.844, 95% CI 1.333-6.069 and p = 0.007), rs1012068 (OR = 2.793, 95% CI 1.316-5.928 and p = 0.01), age (OR = 1.029, 95% CI 1.001-1.057 and p = 0.041) and HCV genotypes (OR = 0.247, 95% CI 0.097-0.630 and p = 0.003) were independently associated with end-stage liver disease complications including cirrhosis and HCC (Table 9).

**Table 9:** **Multiple logistic regression analysis among chronic HCV-infected patients and patients diagnosed with cirrhosis and hepatocellular carcinoma**

Variable	Odds ratio	95% C.I.	p value
Age	1.029	(1.001-1.057)	0.041
Sex	0.979	(0.452-2.121)	0.957
BMI	0.967	(0.911-1.026)	0.265
Platelets	0.995	(0.991-0.999)	0.018
DM2	0.619	(0.277-1.386)	0.244
Genotypes	0.247	(0.097-0.630)	0.003
rs5998152	2.844	(1.333-6.069)	0.007
**Variable**	**Odds ratio**	**95% C.I.**	**p value**
Age	1.03	(1.002-1.058)	0.037
Sex	1.001	(0.464 -2.162)	0.998
BMI	0.967	(0.910-1.026)	0.264
Platelets	0.995	(0.991-0.999)	0.025
DM2	0.642	(0.289-1.426)	0.276
Genotypes	0.243	(0.096-0.614)	0.003
rs1012068	2.793	(1.316-5.928)	0.01

## Discussion

It is well established that the outcome of HCV infection is somewhat influenced by host factors such as advanced age, male gender, obesity and host genetics [26]. Based on this, there is a growing interest in the identification of host genetic variations that may also play a role in the host response to infection and the clinical outcome of the disease. Recently several GWAS studies have been conducted on chronic HCV-infected patients diagnosed with fibrosis, cirrhosis and/or HCC [13],[16],[27]. These studies were conducted in order to help predict clinical outcome and guide management of HCV-infected patients. These studies identified promising prognostic genetic factors including variations in DEPDC5 gene that were linked to HCC development in HCV-infected patients. SNPs rs1012068 and rs5998152 in DEPDC5 gene were described as genetic markers for HCC progression among chronic HCV-infected Japanese patients [16].

In the present study, chronic HCV-infected patients were stratified according to disease progression. SNPs rs1012068 and rs5998152 were genotyped to assess their frequency as genetic susceptibility factors for end-stage liver disease progression in HCV-infected Saudi Arabian population. We have shown that the dominant allele T frequency of both SNPs rs1012068 and rs5998152 was strongly associated with severe outcome of chronic HCV infection and could be considered as a genetic susceptibility factor for HCV infection and for end-stage liver disease progression.

We have also determined that MAF of both SNPs is nearly 30% in Saudi subjects who were included in this study. Other studies have reported 12% of rs1012068 MAF in Japanese population [16], 23% in Chinese population and 26% in Western Europeans as documented in the NCBI database. This variation of MAF determined in some ethnic populations indicates the necessity to conduct genetic polymorphisms with clinical association studies to validate any potential prognostic factor demonstrated in other ethnic population. This study also demonstrates that the frequency of the minor alleles showed a significant difference in the distribution between healthy control subjects and HCV-infected patients. Both alleles, G of rs1012068 and C of rs5998152, were more frequent in the healthy individuals than in the patients, suggesting that they might contribute to protection against HCV infection. However, among chronic HCV-infected Saudi Arabian patients in early- and end-stage liver disease, we did not confirm the association of the risk allele as “G” for DEPDC5 SNP rs1012068 for HCC progression as demonstrated in Japanese population [16] or even for low fibrosis stages [27]. Interestingly, in the current study the patients carrying the heterozygous GT genotype for SNP rs1012068 or CT genotype for rs5998152 were more likely to be cirrhotic or with HCC as compared to those without. However, none of the homozygous genotypes showed a significant distribution in any of the patients’ groups. In contrast to the GWAS conducted by Miki and colleagues on a Japanese population, we have shown a significant difference between chronically infected patients compared to cirr/HCC patients under the dominant model for rs1012068. Our results are in agreement with Miki *et al.* [16] with regard to the dominant model of rs1012068. Such differences suggest that results from these studies are population-dependent. Moreover, the exact definition of the pathological status associated with HCV infection could vary between different groups. To avoid such controversies, it is obvious that studies of genetic susceptibility factors for HCV infection and its sequelae ought to be conducted according to a rigorous clinical stratification of a large cohort of well-defined patients.

Evidence suggests that HCV increases the risk of development of DM2 [28]. It has been reported that the incidence of DM2 to the non-cirrhotic HCV patients is 33% as compared to a control group with no liver disease (5.6%) [29]. In the present study, about a third of HCV patients had DM2, similar to the earlier report. However, another study [30] from the southern part of Saudi Arabia showed a much lower prevalence (15.2%), and there was no significant difference in the prevalence of DM2 between HCC and cirrhosis patients. Factors that could account for the lower prevalence of DM2 may in part be related to differences in age, a family history, dietary habits, obesity/steatosis, or mainly due to inclusion of only advanced liver disease patients (liver cirrhosis and HCC). Moreover, the prevalence of DM between the three subgroups of HCV patients (Chronic HCV, Cirrhotic and HCC) was not statistically significant.

In the present study, we also observed an association between rs1012068 and rs5998152 haplotypes and HCV infection. A significant correlation between the frequency of several haplotypes and end-stage liver complications was evident, demonstrating their potential usefulness as prognostic genetic factors in susceptibility and progression of chronic HCV infection. However, this correlation should be confirmed in a different population and in a larger sample size.

The contribution and the significance of the genetic effects of rs1012068 and rs5998152 SNPs on the molecular mechanisms of action of DEPDC5 are not known, as there are no functional studies on these polymorphisms available, and deserve further investigations at the cellular level. Recently, a study has demonstrated that DEPDC5 mutation was involved in focal epilepsy, a neurodegenerative disease. A GWAS has demonstrated that patients co-infected with HCV and HIV developed more neurologic disorders than HIV mono-infected patients [31]. In addition, HCV has a high mutation rate, which is consistent with the high degree of HCV genetic diversity found across the population of infected individuals. Indeed, HCV is highly variable, with multiple genotypes, and a global genetic diversity that is higher than that of human immunodeficiency virus [32]. This genetic diversity allows fast evolution and escapes from immune and antiviral drug pressure, and may contribute to HCV pathogenesis. Thus far, the only cell function of DEPDC5 mentioned was to block mTOR pathway [18]. A recent study using HCV-infected hepatocytes has described the activation of mTOR signaling induced by HCV through NS5A viral protein [33]. Furthermore, it has been reported that inhibition of mTOR blocks the anti-inflammatory effects of glucocorticoids in myeloid immune cells and might promote fibrosis by a TGF-β-independent mechanism [20],[34]. Altogether, it would be of interest to study DEPDC5 protein function from chronic HCV-infected patients diagnosed with end-stage liver disease to know whether the protein is functional and to investigate its function in the immune system and in hepatocarcinogenesis.

## Conclusions

We have demonstrated that DEPDC5 SNPs, rs1012068 and rs599815were associated with chronic HCV infection and with end-stage liver disease progression. Further investigation is needed to determine the function of DEPDC5 in inflammatory process and eventually in hepatocarcinogenesis.
